# CreA-mediated repression of gene expression occurs at low monosaccharide levels during fungal plant biomass conversion in a time and substrate dependent manner

**DOI:** 10.1016/j.tcsw.2021.100050

**Published:** 2021-03-03

**Authors:** Mao Peng, Claire Khosravi, Ronnie J.M. Lubbers, Roland S. Kun, Maria Victoria Aguilar Pontes, Evy Battaglia, Cindy Chen, Sacha Dalhuijsen, Paul Daly, Anna Lipzen, Vivian Ng, Juying Yan, Mei Wang, Jaap Visser, Igor V. Grigoriev, Miia R. Mäkelä, Ronald P. de Vries

**Affiliations:** a Fungal Physiology, Westerdijk Fungal Biodiversity Institute, & Fungal Molecular Physiology, Utrecht University, Uppsalalaan 8, 3584 CT, Utrecht, The Netherlands; b USA Department of Energy Joint Genome Institute, Lawrence Berkeley National Laboratory, 1 Cyclotron Rd, Berkeley, CA 94720, United States; c Microbiology, Utrecht University, Padualaan 8, 3584 CH Utrecht, The Netherlands; d Department of Plant and Microbial Biology, University of California Berkeley, 111 Koshland Hall, Berkeley, CA 94720, USA; e Department of Microbiology, University of Helsinki, Viikinkaari 9, Helsinki, Finland

**Keywords:** *creA*, Carbon catabolite repression, Transcription factor, *Aspergillus niger*, Fungal plant biomass conversion

## Abstract

Carbon catabolite repression enables fungi to utilize the most favourable carbon source in the environment, and is mediated by a key regulator, CreA, in most fungi. CreA-mediated regulation has mainly been studied at high monosaccharide concentrations, an uncommon situation in most natural biotopes. In nature, many fungi rely on plant biomass as their major carbon source by producing enzymes to degrade plant cell wall polysaccharides into metabolizable sugars. To determine the role of CreA when fungi grow in more natural conditions and in particular with respect to degradation and conversion of plant cell walls, we compared transcriptomes of a *creA* deletion and reference strain of the ascomycete *Aspergillus niger* during growth on sugar beet pulp and wheat bran. Transcriptomics, extracellular sugar concentrations and growth profiling of *A. niger* on a variety of carbon sources, revealed that also under conditions with low concentrations of free monosaccharides, CreA has a major effect on gene expression in a strong time and substrate composition dependent manner. In addition, we compared the CreA regulon from five fungi during their growth on crude plant biomass or cellulose. It showed that CreA commonly regulated genes related to carbon metabolism, sugar transport and plant cell wall degrading enzymes across different species. We therefore conclude that CreA has a crucial role for fungi also in adapting to low sugar concentrations as occurring in their natural biotopes, which is supported by the presence of CreA orthologs in nearly all fungi.

## 1 Introduction

In nature, many filamentous fungi use plant biomass as their main carbon source. They produce extensive sets of extracellular enzymes to be able to penetrate and degrade the polymeric components of plant cell walls into metabolizable sugars and other mono- and oligomers. This ability is controlled at the transcriptional level by a diverse set of transcription factors (TFs) regulating the specificity and efficiency of fungal lignocellulose conversion (Benocci et al., 2017). There is little conservation with respect to the presence of transcriptional activators involved in plant biomass conversion across the fungal kingdom, suggesting a highly diverse approach to the activation of this process. In contrast, the presence of the main regulatory repressor, CreA, is almost fully conserved across the fungal kingdom (Todd et al., 2014) (Suppl. Fig. S1), which implies a highly important role for this regulator, irrespective of fungal lifestyle or biotope.

In the ascomycete fungus *Aspergillus nidulans*, mutations with an altered response to carbon catabolic repression (CCR) were isolated and mapped to *creA* as early as 1975 (Bailey and Arst, 1975). The *creA* gene and its function to regulate CCR in *A. nidulans* were characterized in 1991 (Dowzer and Kelly, 1991). CreA contains two C2H2 zinc finger protein domains that bind to a 5′-SYGGRG-3′ motif (Kulmburg et al., 1993), preferably at two adjacent sites (Cubero and Scazzocchio, 1994) within the upstream regulatory region (URR) of its target genes. It has been suggested to affect a broad range of biological activities, i.e. fungal extracellular enzyme production (Antoniêto et al., 2014, de Vries et al., 1999, Mello-de-Sousa et al., 2014, Rassinger et al., 2018, Sun and Glass, 2011, Tamayo et al., 2008), regulation of carbon and nitrogen metabolism (Cubero et al., 2000, Mathieu and Felenbok, 1994, Prathumpai et al., 2004, Ries et al., 2016), secondary metabolism (Cepeda-García et al., 2014) and even disease progression of human fungal pathogens (Beattie et al., 2017). Mutation of *creA* can result in impaired morphology of fungal colonies in *A. nidulans* (Dowzer and Kelly, 1991), and decrease of growth rates and spore production in *A. niger* (Ruijter et al., 1997).

Previous studies have greatly enhanced our understanding of CreA-mediated regulation, but mainly focused on the role of CreA during fungal growth at high monosaccharide concentrations, e.g. in the presence of 1–3% d-glucose (Prathumpai et al., 2004, Tamayo et al., 2008). This is clearly different from most fungal natural biotopes, in which crude plant lignocellulose with low free sugar content is the predominant carbon source. In addition, studies in the industrial workhorse *Aspergillus niger* demonstrated that repression through CreA is not restricted to d-glucose, but occurs for all monomeric sugars, albeit at different levels (de Vries et al., 2002b), and that this repression is concentration dependent (de Vries et al., 1999). Considering that CreA is one of the few TFs highly conserved across the whole fungal kingdom (Benocci et al., 2017, Todd et al., 2014) (Suppl. Fig. S1) and most fungi rarely experience high concentration of free sugar in their habitat, we hypothesize that CreA also plays a role at low sugar concentrations. However, its influence on fungal adaption to their natural environment has not been addressed so far. Detailed investigations on CreA regulation during fungal growth on plant biomass are essential to enhance our understanding of the biological function of CreA in natural biotopes and may provide new leads for the genetic engineering of fungi towards more efficient cell factories.

In this study, we compared transcriptomic profiles of an *A. niger creA* deletion (Δ*creA*) and reference strain CBS137562 grown on two plant biomass substrates, sugar beet pulp (SBP) and wheat bran (WB), at three time points (4, 24 and 48 h). Both WB and SBP have a very low free sugar level (around 0.02% in WB and 0.004% in SBP, as detected in this study, Supplementary Table S1), but differ significantly in their polysaccharide composition (Benoit et al., 2015). WB contains more d-xylose and d-glucose, and consists mainly of cellulose and (arabino)xylan, while SBP is more abundant in d-galacturonic acid, l-arabinose and d-galactose, and contains cellulose, pectin and xyloglucan as its main polysaccharides. Through systematic comparison of the reference and *creA* deletion strain, we demonstrated that CreA significantly affects many important aspects of plant biomass conversion, including production of carbohydrate active enzymes (CAZymes), sugar transport and sugar catabolism, at significantly lower free sugar levels than previously reported. However, this regulation is strongly carbon source dependent and predominantly occurs at the early stage of fungal growth on lignocellulose. The further comparison of CreA regulon between *A. niger* and *A. nidulans* during their growth on crude plant biomass, and the previously identified cellulose-related CreA/CRE1 regulons in *Trichoderma reesei* (Antonieto et al., 2014), *Penicillium oxalicum* (Li et al., 2015), and *Neurospora crassa* (Sun and Glass, 2011, Wu et al., 2020) showed that a common set of genes were regulated by CreA across different species. In addition, the analysis of binding sites of different TFs in promoter sequences of CreA-regulated genes revealed potential co-regulation between CreA and other important lignocellulose and starch degradation related TFs, such as XlnR (de Vries et al., 2002a, van Peij et al., 1998), AraR (Battaglia et al., 2014, Ishikawa et al., 2018), RhaR (Kowalczyk et al., 2017), GaaR (Alazi et al., 2016, Martens-Uzunova and Schaap, 2008) and AmyR (Tani et al., 2001) associated with cellulose, hemicellulose, pectin and starch degradation.

## 2 Materials and methods

### 2.1 Strains, media and growth conditions

The *A. niger* reference strain CBS137562 (NW249, *cspA1, nicA1, leuA1,* Δ*argB, pyrA6*) (Jalving et al., 2000) and *creA* mutant CBS137561 (*cspA1, nicA1, leuA1,* Δ*argB, pyrA6,* Δ*creA::A. oryzae pyrG*) were used in this study. For the production of spores, Complete Medium (CM) was used (de Vries et al., 2004) with 1% fructose (w/v) and 1.5% (w/v) agar supplemented with 1 mg L^−1^ nicotinamide, 0.2 g L^−1^ leucine, 0.2 g L^−1^ arginine and 1.22 g L^−1^ uridine and strains were incubated for 5 days at 30 °C. Fresh spores were harvested with 10 mL ACES (*N-*(2-acetamido)-2-aminoethanesulfonic acid) buffer. For the transfer experiment, 10^6^ spores mL^−1^ were inoculated in 1 L Erlenmeyer flasks containing 250 mL CM with 2% fructose, including the previously mentioned supplements, and incubated at 30 °C, 250 rpm, for 16 h. Mycelium was then filtered, washed with Minimal Medium (MM) (de Vries et al., 2004) and transferred to MM containing 1% sugar beet pulp (SBP) (w/v) or 1% wheat bran (WB) (w/v) and the previously mentioned supplements. The mycelium was harvested by vacuum filtration and samples were taken after 4 h, 24 h and 48 h of growth. Mycelial samples were dried between tissue papers and immediately frozen in liquid nitrogen. All the experiments were performed in triplicate.

### 2.2 Measurement of extracellular free sugars

To measure the released sugars from SBP and WB by *A. niger* NW249 and the Δ*creA* mutant, 10^6^ spores mL^−1^ were pre-grown in 1 L Erlenmeyer flasks containing 250 mL CM with 2% fructose, including the previously mentioned supplements, and incubated at 30 °C while shaking at 250 rpm, for 16 h. Mycelium was then filtered, washed with MM and transferred to 250 mL Erlenmeyer flask containing 50 mL MM with 1% SBP (w/v) or 1% WB (w/v) and the previously mentioned supplements and were incubated at 30 °C, 250 rpm. Before adding the mycelia to the flasks, 2 mL medium was taken and used as 0 h time point sample. After 4 h, 24 h and 48 h, 2 mL samples were collected and centrifuged for 10 min at 16.000×*g* to remove the mycelia and WB or SBP particles. The supernatant was diluted 10-fold in water before High Performance Liquid Chromatography (HPLC) analysis. Sugar analysis was performed on a Thermo Scientific Dionex ICS-5000^+^ HPLC-PAD system containing a Dionex CarboPac PA1 2 × 50 mm as guard column and Dionex CarboPac PA1 2 × 250 mm as main column with a multi-step gradient of water, 100 mM NaOH and 100 mM NaOH with 1 M sodium acetate as previously described (Mäkelä et al., 2016).

### 2.3 RNA sequencing and read mapping

The transcriptomes of the reference strain and the *creA* mutant induced on different carbon sources were analysed using RNA sequencing (RNA-seq). RNA was extracted from ground mycelia using TRIzol® reagent (Invitrogen, Breda, the Netherlands) and purified with NucleoSpin® RNA II Clean-up kit (Macherey-Nagel) with rDNase treatment. The RNA quantity and quality was checked with a RNA6000 Nano Assay using the Agilent 2100 Bioanalyzer (Agilent Technologies, Santa Clara, CA, USA). Purification of mRNA, synthesis of cDNA library and sequencing were conducted at Joint Genome Institute (JGI). RNA samples were single-end sequenced using Illumina HiSeq 2000/2500 platform (http://illumina.com). Raw fastq file reads were filtered and trimmed using the JGI QC pipeline. Using BBDuk (https://sourceforge.net/projects/bbmap/) raw reads were evaluated for artefact sequence by kmer matching (kmer = 25), allowing one mismatch and detected artefact was trimmed from the 3′ end of the reads. RNA spike-in reads, PhiX reads and reads containing any Ns were removed. Quality trimming was performed using the phred trimming method set at Q6. Reads under the length threshold were removed. Three individual samples with poor sequencing quality were discarded from further analysis. The cleaned reads were mapped to *A. niger* NRRL 3 transcript and counted with Salmon (Patro et al., 2017). We refer to *A. niger* NRRL 3 genome IDs based on the most up-to-date and accurate annotation of the *A. niger* NRRL 3 genome (http://genome.fungalgenomics.ca/) (Aguilar-Pontes et al., 2018).

### 2.4 RNA-seq data analysis

DESeq2 v1.20 (Love et al., 2014) was used to determine differentially expressed genes (DEGs) between Δ*creA* and the reference strain in each conditions. Raw counts were used as DESeq2 input. The threshold of fold change ≥4 and adjusted P-value <0.01 was used to define significant differentially expressed genes (DEGs). The gene expression level was measured as Fragments Per Kilobase of transcript per Million mapped reads (FPKM) (Trapnell et al., 2010). Transcriptomic analysis mainly focused on genes encoding plant cell wall degradation (PCWD) related CAZymes, carbon metabolic enzymes, sugar transporters and transcription factors (TFs). Sugar transporter genes and their phylogenetic analysis were derived from (Peng et al., 2018a). Carbon metabolic enzymes and pathways were retrieved from (Aguilar-Pontes et al., 2018). The PCWD CAZy substrate and enzyme annotations were based on (Benoit et al., 2015, Gruben et al., 2017). Unless stated otherwise, all transporter or enzyme functions are putative.

### 2.5 Gene Ontology analysis and data visualization

The Gene Ontology (GO) annotation was retrieved from JGI MycoCosm database (https://genome.jgi.doe.gov/Aspni_NRRL3_1/Aspni_NRRL3_1.home.html) and the Gene Ontology (GO) annotation database (Carlson, 2018) from R Bioconductor was used to map their ancestor nodes in the GO hierarchy. The GO Slim terms defined in AspGD (http://www.aspgd.org/) were selected for enrichment analysis. The GO terms enriched within the significant differentially expressed gene lists compared to the genome background were detected by a hypergeometric distribution model calculated with in-house script. The statistical analysis was performed with R v3.5.1 software platform. The P-values for multiple tests were corrected with Benjamini and Hochberg's method (Benjamini and Hochberg, 1995).

Heatmap and clustering were plotted using R package “ggplot2” using the parameters of Euclidean distance and complete linkage method. The principal component analysis (PCA) was performed with R package “FactoMineR”. Pathway graphs were manually created using Adobe Illustrator.

### 2.6 Transcription factor binding site analysis

The 1000 bp length of promoter sequences upstream of the coding region of the genes were obtained from JGI MycoCosm database (https://genome.jgi.doe.gov/Aspni_NRRL3_1/Aspni_NRRL3_1.home.html). The reported CreA binding motif 5′-SYGGRG-3′ and its sub-motifs 5′-SYGGGG-3′ and 5′SYGGAG-3′ were searched in both strands of promoter sequences as in a previous study (Portnoy et al., 2011) using an in-house Perl script. The definition of adjacent motifs pair was restricted to motifs within 100 bp of each other in the promoter sequences of targeting gene. Similarly, the reported binding motif 5′-GGCTA[AG]-3′ for XlnR (de Vries et al., 2002a, van Peij et al., 1998), 5′-CGG[AGT]TAA[AT]-3′ for AraR (Battaglia et al., 2014, Ishikawa et al., 2018), 5′-TG[CAG][GTA]GGG-3′ for RhaR (Kowalczyk et al., 2017), 5′-CC[ACGT]CCAA-3′ for GaaR (Martens-Uzunova and Schaap, 2008) and 5′-CGGN_8_CGG-3′ for AmyR (Ito et al., 2004, Tani et al., 2001) were searched in promoter sequences. The enrichment of specific TF binding sites distributed in promoters of Δ*creA*-regulated genes compared to their distribution in the genome level was calculated with the ‘fisher.test’ function in R.

### 2.7 Phylogenetic analysis

Amino acid sequences of the CreA homologs across the fungal kingdom were retrieved from a previous study (Benocci et al., 2017). Sequence alignment was performed with MAFFT (Katoh and Standley, 2013). MEGA software (version 7.0) was used for the phylogenetic analysis (Kumar et al., 2016). Phylogenetic trees based on the Maximum Likelihood, Neighbor Joining and Minimal Evolution algorithms were built separately using 500 bootstraps.

### 2.8 Comparative transcriptome analysis of the CreA regulon

The CreA regulon of *A. niger* identified in this study was compared to the CreA regulon reported in several other fungi. Transcriptome data of the *A. nidulans creA* mutant and reference strain grown on WB was obtained from our previous study that was originally used to investigate the CreA effects on sugar catabolism (Khosravi et al., 2018). This dataset (GEO accession: GSE94775) was reanalysed to identify the genome-scale CreA regulon. The CreA regulon of *T. reesei* grown on cellulose and glucose was extracted from a previous study (Antonieto et al., 2014), as was the CreA regulon of *P. oxalicum* on cellulose (Li et al., 2015, GEO accession: GSE69298). The CreA regulon of *N. crassa* was directly obtained from two independent studies. One study compared the *creA* mutant grown on cellulose and minimal media using microarray (Sun and Glass, 2011) and the other one identified CreA binding genes using DNA affinity purification sequencing (DAP-seq) technology (Wu et al., 2020). Except that the original reported regulons of *N. crassa* were used in the comparison, we used the DEseq2 R package (Love et al., 2014) and the same threshold to detect the differentially expressed genes in other species since the RNA-seq was commonly used in these studies for identification of the CreA regulon. Intersecting sets among different regulons were visualized with the R package UpSetR (Conway et al., 2017). GO analysis was performed similarly as described in the above *A. niger* analysis.

The predicted proteome of each studied fungus was downloaded from the JGI MycoCosm database (Grigoriev et al., 2014) and used for an all-against-all BLAST comparison with subsequent clustering of orthologous genes. The OrthoMCL method was used to infer orthologous genes across different species (Li et al., 2003) with parameters set as follows: E-value 1E−5, inflation level 1.5 and sequence coverage 60%. The OrthoMCL algorithm generates clusters of proteins where each cluster consists of orthologs or “recent” paralogs across multiple fungal species. Comparison of the CreA regulon across different species was based on gene expression changes between the *creA* mutant and reference strain in each protein cluster.

## 3 Results

### 3.1 Transcriptomics reveals a major role for CreA during growth on plant biomass

**Fig. 1:**
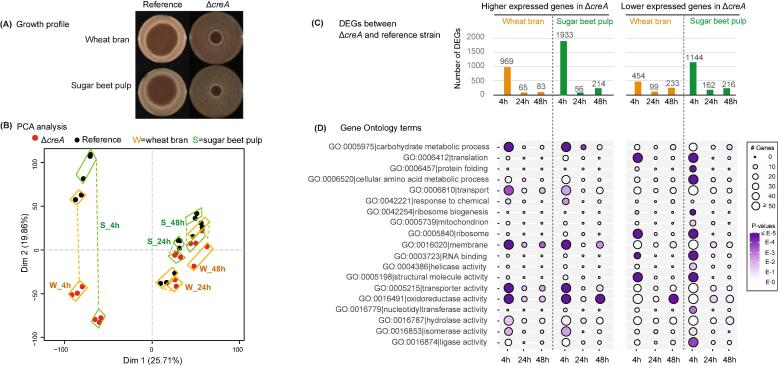
Global transcriptome profile of the *A. niger* Δ*creA* and reference strain. (A) Comparison of the growth of *A. niger* reference and Δ*creA* strain on WB and SBP. Strains were inoculated with 1000 spores in 2 µl and grown for five days at 30 °C. Crude carbon sources were added at 3% (w/v) final concentration. (B) PCA analysis performed with expression values (FPKMs) of all detected genes. (C) The numbers of higher and lower expressed genes detected in comparison between the Δ*creA* and reference strain. (D) Gene Ontology (GO) analysis of higher and lower expressed genes in the *A. niger* Δ*creA* strain compared to the reference strain. The size and color of the circle on the figure indicate the gene number and statistical significance of the enriched GO terms.

CreA is one of the few TFs that is broadly conserved across the fungal kingdom (Todd et al., 2014) (Supplementary Fig. S1 and Table S2). The extreme conservation of CreA suggests an important role in fungal adaption to its natural environment. The growth profile of *A. niger creA* deletion (Δ*creA*) and reference strain on two crude plant biomass (SBP and WB), showed that the Δ*creA* mutant tends to grow slowly and with reduced sporulation on both substrates (Fig. 1A). To further investigate the CreA mediated regulation mechanism of plant biomass conversion, RNA-seq experiments were performed to compare transcriptomic changes between Δ*creA* and the reference strain grown on SBP and WB. Our study demonstrated that the low free monosaccharide levels present in these substrates already activate CreA, therefore indicating a clear role for this repressor protein during growth of *A. niger* on plant biomass. The comparison of global transcriptome profiles between the *A. niger* Δ*creA* mutant and the reference strain revealed that CreA-mediated regulation of lignocellulose conversion is strongly carbon source and time dependent (Fig. 1B–D). PCA analysis showed that the largest clustering distance between the strains was observed at the early time point (4 h) for both WB and SBP (Fig. 1B), indicating that the strongest CreA effect occurred at the early stage of lignocellulose conversion. In addition, the clustering distance between the reference strain on SBP and WB at 4 h was smaller than their distance to the corresponding Δ*creA* samples, suggesting that the deletion of *creA* caused more transcriptome level changes than the different carbon sources at this time point. CreA-mediated regulation was less pronounced at the later time points of the cultivation, as indicated by the close clustering of the reference and Δ*creA* strain (Fig. 1B).

**Fig. 2:**
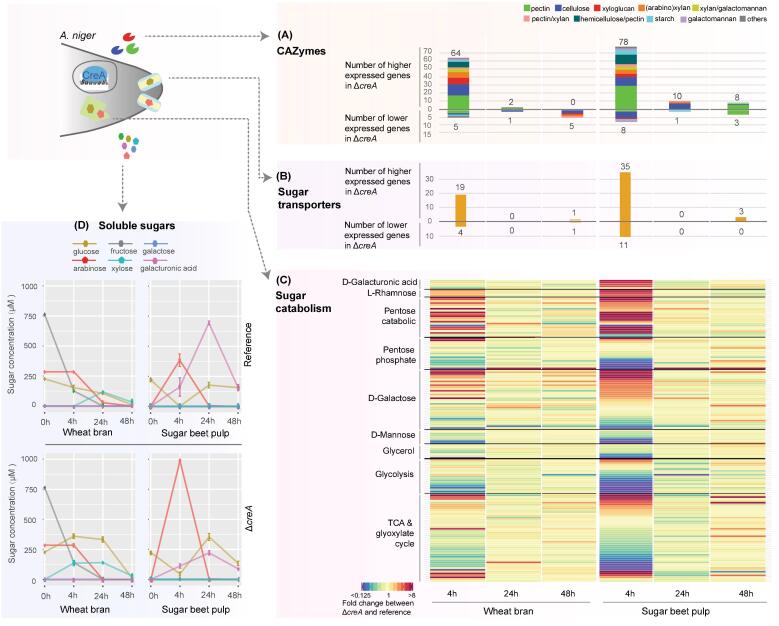
Transcriptomic changes of key plant biomass degrading activities between *A. niger* Δ*creA* and reference strain cultivated on wheat bran and sugar beet pulp for 4, 24 and 48 h. (A) Differentially expressed PCWD related CAZymes detected in comparison between the *A. niger* Δ*creA* and reference strain. CAZymes were categorized according to their specific polysaccharide substrates and were visualized with different colors on the bar chart. (B) Differentially expressed sugar transporters encoding genes in comparison between *A. niger* Δ*creA* and reference strain. (C) Transcriptomic changes of sugar catabolic genes between *A. niger* Δ*creA* and reference strain. The color from blue to red indicate genes from lower to higher expression, respectively, in Δ*creA* compared to the reference strain. (D) The concentration profile of extracellular sugars detected from the cultivations. The error bars represent the standard error of the mean. (For interpretation of the references to color in this figure legend, the reader is referred to the web version of this article.)

The *A. niger* strains showed a similar dynamic trend between the number of differentially expressed genes (DEGs) and the global transcriptome clustering results (Fig. 1C and Supplementary Table S3). When the Δ*creA* mutant was compared to the reference strain, for both WB and SBP, the highest number of DEGs (1423 and 3077 genes, around 12% and 26% of the total encoding genes in the genome, respectively) was detected at the early time point (4 h), whereas a much smaller number of DEGs (164 and 316 genes for WB at 24 h and 48 h, and 218 and 430 genes for SBP at 24 h and 48 h, respectively) was present at later time points. In addition, more than twice as many DEGs were detected in the SBP compared to the WB cultures (Fig. 1C), which was partially caused by the higher abundance of pectin in the SBP that induced a wide array of genes for conversion of this complex polysaccharide (Supplementary Table S3) (Voragen et al., 2009). The dramatic decrease of the number of DEGs at 24 h and 48 h compared to 4 h indicates that the influence of CreA on the fungal response to lignocellulose reduces at later stages of plant biomass conversion. This is consistent with the low concentration of extracellular repressing monosaccharides, ranging from 252 µM to 44 µM, detected at the later time points, compared to 566 µM and 384 µM detected on WB and SBP, respectively, at 4 h (Fig. 2D).

Gene Ontology (GO) enrichment analysis showed that the deletion of *creA* affected a broad range of biological activities during growth of *A. niger* on plant biomass (Fig. 1D). For the higher expressed genes in Δ*creA* compared to the reference strain, several GO terms were commonly enriched on both WB and SBP at 4 h, such as carbohydrate metabolic process, transport activity, oxidoreductase activity, hydrolase activity, isomerase activity and membrane. For the lower expressed genes in Δ*creA*, several GO terms were also commonly enriched during the early stage of lignocellulose conversion (Fig. 1D), including protein translation, structural molecule activity, RNA binding and ribosome. In addition, the GO terms of protein folding, cellular amino acid metabolic process, and ligase activity were uniquely enriched in Δ*creA* on SBP at 4 h, suggesting that protein degradation (de Assis et al., 2018) could be associated with CreA regulation. The GO term for oxidoreductase showed a more diverse enrichment pattern which was not only enriched in the higher expressed genes of Δ*creA* on WB at 4 h, and on SBP at 4 h and 48 h, but also clearly enriched in lower expressed genes of Δ*creA* on WB at 48 h. These differences in GO functions between the *A. niger* Δ*creA* and reference strain demonstrate that the influence of CreA goes far beyond the classic definition of carbon catabolite repression (CCR) (Ruijter and Visser, 1997) during fungal plant biomass conversion.

### 3.2 Polysaccharide degradation, sugar transport and sugar catabolism are tightly regulated by CreA

The above GO analysis revealed that several key fungal plant biomass conversion related activities (Antoniêto et al., 2014, Peng et al., 2018b, Sun and Glass, 2011) are significantly affected by CreA, including polysaccharide degradation, sugar transport and sugar catabolism, despite the low free monosaccharide levels present in the substrates. We therefore analysed the expression profiles of these genes in more detail. Of the 203 PCWD-related CAZymes encoding genes predicted in the *A. niger* genome (Benoit et al., 2015, Gruben et al., 2017, Kowalczyk et al., 2017), 101 genes were significantly up-regulated in Δ*creA* compared to the reference strain in at least one of the tested conditions. Especially at the 4 h time point on WB and SBP a high number (64 and 78, respectively) of CAZy genes were upregulated in Δ*creA*, with the highest proportion of them encoding enzymes acting on pectin and cellulose (Fig. 2A and Supplementary Table S3). In addition, many CAZy genes that were only induced at later time points (24 h and 48 h) in the reference strain were highly expressed at the early time point (4 h) in Δ*creA* (Supplementary Fig. S2). This indicates that CreA plays a crucial role in controlling the induction level of CAZy genes at the early stage of fungal growth on lignocellulose. Consistent with SBP being rich in pectin, more pectin-related CAZy genes were significantly higher expressed in Δ*creA* on SBP (31 genes) than on WB (18 genes) (Fig. 2A). The different expression patterns of CAZy genes between the Δ*creA* and reference strains on the two tested plant biomasses suggest that the expression of CAZy genes is co-regulated by CreA-mediated repression and substrate-specific induction controlled by several transcriptional activators. The expression of the genes encoding these transcriptional activators is also affected by CreA. For instance, the pectin conversion related TFs encoding *gaaR* (NRRL3_8195) and *rhaR* (NRRL3_1496) were 5.2 and 1.8 times upregulated, respectively, in Δ*creA* on SBP at 4 h compared to the reference (Supplementary Table S3). Therefore, part of the increased expression of pectinolytic genes in Δ*creA* could be an indirect effect of overexpression of the pectinolytic activators.

Sugar transport is another essential part of the fungal plant biomass conversion process. Sugar transporters (STs) take up the mono- or short oligosaccharides resulting from extracellular enzymatic degradation of lignocellulosic polysaccharides, and transport them into the cell for carbon metabolism. Our recent study predicted 86 STs and their sugar specificity in *A. niger* (Peng et al., 2018a). In total, 47% (40 genes) of the STs were significantly higher expressed in *A. niger* Δ*creA* in at least one of the studied conditions, which indicates a significant effect of CreA on sugar uptake (Fig. 2B and Supplementary Table S3). Of all the ST encoding genes that were differentially expressed between the strains, most were only up-regulated at the early time point in both WB and SBP. The expression of two well-characterized STs showed good correlation to the different composition of the plant biomass substrates (Supplementary Fig. S3). The d-galacturonic acid transporter encoding gene *gatA* (NRRL3_958) was higher expressed on SBP, where it was most significantly up-regulated at 4 h. In contrast, the d-xylose transporter encoding gene *xltA* (NRRL3_11715) was higher expressed on WB and was most significantly up-regulated at 4 h.

*A. niger* is able to utilize a diverse set of monosaccharides through a diverse set of sugar metabolic pathways (Aguilar-Pontes et al., 2018). The deletion of *creA* significantly changed the expression of several sugar catabolic pathways (Fig. 2C and Supplementary Table S3) and the transcriptome profiles of these pathways showed strong carbon source specificity (Supplementary Fig. S4). For the pentose catabolic pathway, most of the genes were significantly higher expressed in Δ*creA* grown on both SBP and WB at 4 h compared to the reference strain. A stronger de-repression of l-arabinose conversion related genes *larA*, *ladA*, *ladB* and *lxrA* was observed for Δ*creA* grown on SBP than on WB, while the d-xylose reductase *xyrA* was more significantly de-repressed in Δ*creA* grown on WB than on SBP. This could be explained by higher abundance of l-arabinose and d-xylose in SBP and WB, respectively (Benoit et al., 2015), which caused a different extent of de-repression of the corresponding sugar catabolic genes in Δ*creA* compared to the reference strain. The pentose phosphate pathway and d-galactose catabolism related genes showed more diverse expression profiles, containing both Δ*creA* up- and down-regulated genes. Consistent with higher d-galacturonic acid and l-rhamnose content in SBP than in WB, stronger de-repression of the corresponding carbon catabolic genes were observed in Δ*creA* grown on SBP than on WB (Fig. 2C and Supplementary Fig. S4). In contrast to the above described pathways, expression of most of the glycolytic genes were either down-regulated or not changed in Δ*creA* on both WB and SBP. Since glycolysis is the central pathway of carbohydrate metabolism, the overall down-regulation of glycolytic genes could have resulted from the reduced metabolism and growth of Δ*creA* on both SBP and WB.

### 3.3 Co-regulation between CreA and other TFs in controlling plant biomass conversion

The different expression patterns across the two plant biomass substrates and different time points indicate that CreA acts in concert with specific transcriptional activators to precisely regulate their target genes under different growth conditions. In line with this, several important lignocellulolytic genes were previously reported to be co-regulated by CreA and transcriptional activators. For example, in *A. niger* the expression of the feruloyl esterase encoding gene *faeA* and the α-glucuronidase encoding gene *aguA* has been shown to be controlled by both CreA and the (hemi-)cellulolytic transcriptional activator XlnR (de Vries et al., 1999), while the *exo*-polygalacturonase encoding gene *pgaX* is co-regulated by CreA and the pectinolytic transcriptional activator GaaR (Niu et al., 2015).

To reveal additional genes co-regulated by CreA and other plant biomass conversion related TFs, we analysed the promoter sequences of CreA-regulated genes detected in this study for possible binding sites for CreA and several well-characterized lignocellulose conversion related transcriptional activators, i.e. XlnR (de Vries et al., 2002a, van Peij et al., 1998), AraR (Battaglia et al., 2014, Ishikawa et al., 2018), RhaR (Kowalczyk et al., 2017), GaaR (Alazi et al., 2016, Martens-Uzunova and Schaap, 2008), and AmyR (Ito et al., 2004, Tani et al., 2001). We analysed the frequencies at which two sub-types of the CreA 5′-SYGGRG-3′ binding motif occurred. The occurrence of a single motif 5′-SYGGGG-3′ was 21% higher in Δ*creA* up-regulated genes than its occurrence in the genome. However, the motif 5′-SYGGAG-3′ was neither clearly enriched in CreA targets detected in this study compared to its occurrence in the genome, nor in previously characterized CreA targets (Supplementary Table S4). This suggests that the 5′-SYGGGG-3′ is the functional version of the CreA binding motif in *A. niger*.

Previous studies also indicated that CreA prefers to bind to adjacent 5′-SYGGRG-3′ motifs (Cubero and Scazzocchio, 1994, Mathieu and Felenbok, 1994). In total, 716 (31%) of 2312 up-regulated genes in the Δ*creA* cultivations contain the adjacent 5′-SYGGGG-3′ motif, while only 23% (2760 genes) of all protein encoding genes in the *A. niger* genome contain this adjacent motif pair. This means that the frequency of occurrence for this adjacent motif pair in promoters of Δ*creA* up-regulated genes is 37% higher than its occurrence in the whole genome (Supplementary Table S4).

For the 716 genes containing the adjacent 5′-SYGGGG-3′ CreA binding motif, 45%, 15%, 84%, 47% and 33% of these genes also contain a binding motif for XlnR, AraR, RhaR, GaaR or AmyR, respectively. This is higher than their distribution at the genome level (39%, 9%, 68%, 41% and 27%, respectively) with statistically significant P-values (8E−3, 2E−5, 2E−16, 5E−3 and 1E−4, respectively) according to the Fisher’s exact test (Supplementary Fig. S5). In line with this, 18 important plant biomass conversion genes, including hemicellulolytic, pectinolytic and amylase-encoding genes, and pentose catabolic genes, identified as CreA target genes in this study were previously reported to be regulated by transcriptional activators (Supplementary Table S5). In the promoters of the Δ*creA* down-regulated genes, no enrichment of the reported CreA binding motif was found, which indicates that their reduced expression is likely due to indirect regulatory effects.

The co-regulation by CreA and other lignocellulose conversion TFs is not only suggested by over*-*representation of the co-occurring TF binding sites within promoter regions of their targets, but is also seen from the expression changes of the TF encoding genes in Δ*creA* (Supplementary Fig. S6). Several of these TFs, e.g. *gaaR*, *xlnR*, *manR* and *clrB*, showed some extent of de-repression in the Δ*creA* mutant at the early time point of plant biomass conversion. The de-repression of *rhaR*, *inuR* and *pacC* in the Δ*creA* were mainly observed at 4 h on SBP, while the de-repression of *amyR* occurred at 48 h on both WB and SBP. In contrast, the l-arabinose responsive regulator encoding gene *araR* showed an opposite expression pattern, being down-regulated in Δ*creA*. In addition, the nitrogen-sensing regulator *areA* was up-regulated in the Δ*creA* mutant at the early time point on lignocellulose substrates, which is in line with the previously suggested cross-talk between these two TFs (Wilson and Arst, 1998).

### 3.4 The concentration of extracellular sugars supports the transcriptomic changes in the creA deletion strain

The concentration profile of extracellular sugars varied significantly with respect to *A. niger* strain, plant biomass substrate and incubation time (Fig. 2D and Supplementary Table S1). For the reference strain grown on WB, arabinose, glucose and fructose were the major soluble sugars detected at 4 h with a total concentration of 566 μM. The concentration of arabinose and fructose decreased from 286 μM and 126 μM at 4 h, respectively, to nearly zero at 24 h, while the glucose concentration decreased from 153 μM at 4 h to 106 μM at 24 h. In contrast, the xylose concentration increased from zero at 4 h to 118 μM at 24 h. At 48 h, only a very low level of glucose and xylose was detected (12 μM and 31 μM, respectively). In Δ*creA* grown on WB, the concentration of xylose and glucose at 4 h reached 136 μM and 360 μM, respectively, which was clearly higher than in the reference strain. At later time points, no other significant changes of the sugar profile was observed between the strains, except that the concentration of glucose in Δ*creA* was 3-fold higher at 24 h compared to the reference strain.

For the reference strain grown on SBP, arabinose and galacturonic acid were the major soluble sugars detected at 4 h. The concentration of arabinose decreased from 384 μM at 4 h to nearly zero at later time points. The glucose concentration increased from zero at 4 h to 177 μM at 24 h and slightly decreased to 156 μM at 48 h. The galacturonic acid concentration changed significantly over the cultivation, as it increased from 161 μM at 4 h to 694 μM at 24 h, and decreased to 157 μM at 48 h. In the Δ*creA* strain grown on SBP, extracellular arabinose reached 987 μM at 4 h, which was significantly higher than in the reference strain, while the galacturonic acid at 24 h was present at much lower concentration (221 μM) compared to the reference strain. In addition, we detected that a low level of soluble sugars pre-existed in the crude plant biomass before the fungi were cultivated on them, with a total concentration around 1270 μM (~0.02%) of glucose, arabinose and fructose in WB, and 221 μM (~0.004%) glucose in SBP (Fig. 2D).

Overall, the dynamic profile of soluble sugars concentration correlated well with the dynamic transcriptomic changes discussed above. The total concentration of free monosaccharides (arabinose, glucose and fructose) at 4 h was relatively high compared to 24 h and 48 h, which led to a more significant CreA-mediated regulation of gene expression level at early time point compared to later time points in the reference strain (Fig. 1). The deletion of *creA* alleviated the carbon repression effect at 4 h and increased the release of extracellular soluble sugars, as evidenced from the significant increase of glucose and xylose on WB at 4 h and of arabinose on SBP at 4 h in Δ*creA* compared to the reference strain. The repressing effect of galacturonic acid is weaker than that of the other sugars from WB and SBP in *A. niger* (de Vries et al., 2002b). Therefore, although a relatively high concentration of galacturonic acid was detected at 24 h in the reference strain grown on SBP, a much smaller number of DEGs was detected between the reference strain and Δ*creA* at this time point compared to the 4 h time point. The sugar changes across different growth conditions were consistent with expressional changes of several known sugar transporter genes. For instance, the d-galacturonic acid transporter encoding gene *gatA* (NRRL3_958) was higher expressed on SBP, especially at 24 h in which the d-galacturonic acid concentration was highest compared to other time points. The hexose transporters encoding genes *mstA* (NRRL3_3147) and *mstH* (NRRL3_3879) and pentose transporter encoding gene *xltC* (NRRL3_10052) were higher expressed at 4 h than later time points on both SBP and WB in which higher hexose and pentose concentrations were detected.

### 3.5 Comparative transcriptome revealed that CreA regulated a common set of genes across different species

No specific studies into the effect of CreA at low sugar concentrations have been done before, but other CreA studies can still be used to investigate the conservation of CreA regulation across fungi during plant biomass conversion. Therefore, we compared the CreA regulon of *A. niger* determined in this study (2190 DEGs detected in both WB and SBP at all time points) to CreA regulons identified from four other fungi. These include the CreA regulon of *A. nidulans* identified through re-analysing a transcriptome data of a *creA* mutant and a reference strain grown on wheat bran (Khosravi et al., 2018) and the CreA regulon of *P. oxalicum* identified through comparing the transcriptome of a *creA* mutant and reference during their growth on cellulose (Li et al., 2015). Also, the CreA regulon of *T. reesei* was identified by transcriptomics analysis during growth on cellulose and glucose (Antonieto et al., 2014) whereas the CreA regulon of *N. crassa* was identified by microarray analysis during growth on cellulose and minimal media (Sun and Glass, 2011) and the CreA binding genes were detected through DAP-seq technology (Wu et al., 2020).

**Fig. 3:**
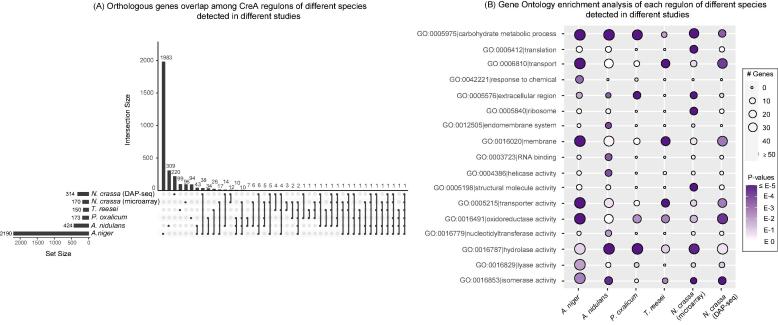
Comparative transcriptome analysis of CreA regulon across different fungal species. (A) CreA target gene overlap across datasets from five different species. The CreA regulon of the *A. niger* was identified in this study, while the CreA regulon of the *A. nidulans* grown on WB was derived from our previous study (Khosravi et al., 2018). The CreA regulon of *T. reesei* grown on cellulose and glucose was extracted from a previous study (Antonieto et al., 2014), as was the CreA regulon of *P. oxalicum* on cellulose (Li et al., 2015). The CreA regulon of *N. crassa* was obtained from two independent studies. One study compared the *creA* mutant grown on cellulose and minimal media using microarray (Sun and Glass, 2011) and the other one identified CreA binding genes using DAP-seq technology (Wu et al., 2020). The OrthoMCL method was used to infer orthologous genes across different species (Li et al., 2003). Intersecting sets among different regulons were visualized with the R package UpSetR (Conway et al., 2017). (B) Gene ontology enrichment analysis of CreA regulon identified from different datasets. The size and color of the circle on the figure indicate the gene number and statistical significance of the enriched GO terms.

Compared to the CreA regulon identified in other fungal species during their growth on crude plant biomass or cellulose at a single time point, we reported a much larger set of CreA target genes in *A. niger*, likely due to the fact that our transcriptome analysis evaluated growth on two crude plant biomasses at multiple time points. Around 27%–46% CreA target genes identified in previous data were shared by more than one species (Fig. 3A). GO enrichment analysis of each of the regulons from different species revealed that several GO terms were commonly enriched in most of the species, including the carbohydrate metabolic process, hydrolase activity, transporter activity and oxidoreductase activity (Fig. 3B).

Several known plant biomass conversion genes found in the *A. niger* CreA regulon were also commonly regulated by *creA* in other fungi (Supplementary Table S6). Specifically, an amylolytic gene (*glaA*) and a cellulolytic gene (*bgl4*) have been identified in the CreA regulon of all five studied species. Several genes were present in the CreA regulon of four species, including the (arabino)xylanolytic genes *axeA*, *xlnB*, *xlnC* and *xlnD*, the cellulolytic genes *cbhA* and *cbhB*, the galactomannanolytic gene *mndB*, the hemicellulolytic/pectinolytic gene *abfB*, the pectinolytic genes *galA*, *pgxB*, *rgaeA*, and *rglA*, the amylolytic gene *agdB*, the cellodextrin transporter encoding gene *ctA*, and the hexose transporter encoding gene *mstA*. In addition, several known sugar catabolic enzyme encoding genes and TF encoding genes related to plant biomass conversion have been identified in the regulon of three of the species, such as the glycolytic gene *pkiA*, the pentose catabolic genes *ladA* and *xkiA*, and the cellulose regulator encoding genes *clrA* and *manR*. In spite of differences in the growth conditions and analysis platforms, the identification of a common set of CreA target genes further supports the conserved role that CreA plays in fungal plant biomass conversion.

## 4 Discussion

Filamentous fungi are commonly considered to rarely experience a high free sugar concentration in their natural biotopes that could induce CCR, as mainly polymeric carbon sources are present and they need to compete with other microorganisms for the available sugars. For example, the soluble sugar concentration detected in forest soils is between 0.0005 and 0.01% (dry weight) (Gunina and Kuzyakov, 2015, Johnson and Pregitzer, 2007), and the highest concentration of free soluble sugar detected in this study was only around 0.02% (dry weight) on WB at 0 h and 0.006% on SBP at 4 h, respectively (Fig. 2D and Supplementary Table S1). These are all much lower than previously reported repressing sugar concentrations for several fungal species (e.g. 1%/56 mM d-glucose (Antoniêto et al., 2014, Fernandez et al., 2012, Prathumpai et al., 2004, Ruijter et al., 1997, Tamayo et al., 2008)). However, we demonstrated that at early stage of plant biomass conversion, *A. niger* transiently experiences significant CCR due to the soluble sugars present in lignocellulose together with those released by the fungus. This indicates that CreA-mediated CCR already occurs at very low sugar concentrations, far below mM levels as was previously shown for various xylose concentrations in *A. niger* (de Vries et al., 1999).

**Fig. 4:**
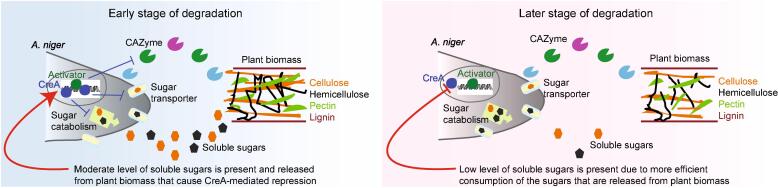
A dynamic model of CreA regulation during plant biomass conversion by *A. niger*. At early stage of the process, the fungus senses the composition of lignocellulose and expresses specific TFs to activate expression of key genes, such as CAZymes, sugar transporters and catabolic enzymes encoding genes, to release and catabolize sugars derived from polysaccharides. When more soluble sugars are released, together with the free sugars that pre-exist in plant biomass, the sugar concentration reaches a level to trigger the CreA-mediated carbon catabolic repression (CCR), which exerts a negative feedback on the process of sugar release and utilization. At later stages of the degradation, due to the more efficient consumption of the sugars, the concentration of extracellular sugars decreases and the CreA repression is alleviated.

Based on the results of our study, a dynamic model of CreA regulation for controlling plant biomass utilization in *A. niger* is proposed (Fig. 4). At the early stage of the degradation, e.g. after 4 h, a small set of CAZymes, produced at low constitutive level, release small amounts of mono- and disaccharides. These sugars are transported into the cell, resulting in the activation of specific TFs. These regulators promote expression of key genes needed to efficiently release, transport and catabolize sugars derived from plant biomass polysaccharides. With the release of more soluble sugars, together with those already present in fungal natural biotopes due to the action of other microorganisms, these sugars transiently reach a concentration that triggers the CreA-mediated CCR. This exerts a negative feedback on the process of sugar release and utilization. At later time points, as consumption of the sugars becomes more efficient, the concentration of extracellular free sugars decreases and the CreA-mediated CCR is alleviated.

In nature, multiple microorganisms live in the same ecological niches. Therefore, this sophisticated regulation prevents lignocellulose degrading fungi from wasting energy on releasing excess sugars from plant biomass beyond their sugar catabolic capacity that would benefit other microorganisms in their habitat. Therefore, we suggest that the CreA-mediated CCR plays an important role in adaptation and competition of fungi in their natural environment, where penetration and degradation of plant cell walls is a common feature for fungi.

Considering the conservation of CreA in the fungal kingdom, it can be postulated that a similar role exists for its homologs in other species. CreA orthologs can be found in nearly all fungi, with the exception of the yeast *S. cerevisiae* and some of its related species (Supplementary Fig. S1 and Table S2) (Adnan et al., 2017, Benocci et al., 2017). Instead, *S. cerevisiae* contains MIG1 (Klein et al., 1998), which is a functional homolog of CreA despite sharing low sequence identity (30%). The broad conservation of CreA across fungi is further supported by the presence of a similar system for CCR in plant pathogenic ascomycete fungi (Tannous et al., 2018, Tudzynski et al., 2000) and in the taxonomically distant basidiomycete fungi (Daly et al., 2019, Hu et al., 2020, Stapleton et al., 2004, Suzuki et al., 2008, Zhang and Schilling, 2017). For example, deletion of the CreA homolog of the white rot basidiomycete *Pleurotus ostreatus* (CRE1) revealed increased cellulase activity and cellulose degradation, as well as increased abundance of a broad set of CAZymes during growth on wheat straw (Yoav et al., 2018).

There is only a limited number of detailed CCR studies on CreA homologs in relation to plant biomass, but they do confirm the similar role, although also point towards species-specific differences. Next to *A. niger*, the best studied species with respect to CCR mediated by CreA orthologs, are the ascomycetes *T. reesei* (Cre1) and *N. crassa* (CRE-1). Analysis of the regulon of Cre1 in *T. reesei* revealed a similar set of target genes as for *A. niger*, including genes encoding CAZymes, sugar transporters and transcriptional regulators (Antonieto et al., 2016, Antoniêto et al., 2014), although analysis of the putative binding site of Cre1 resulted in a consensus sequence that differed from the *Aspergillus* consensus sequence (Silva-Rocha et al., 2014). Classical strain improvement resulted in a hyper-cellulolytic strain RUT-C30 in *T. reesei* (Montenecourt and Eveleigh, 1977). This strain includes a truncation of Cre1, resulting in partial de-repression in the presence of D-glucose (Ilmén et al., 1996). This truncated version and a full deletion of Cre1 were compared in detail, demonstrating that the full deletion results in significant growth deficiencies, such as observed for *A. niger* (Supplementary Fig. S7) and *A. nidulans* (Khosravi et al., 2018, Shroff et al., 1997, Shroff et al., 1996), while the truncated version did not show this. In addition, enrichment of the truncated protein in the promoter of the (hemi-)cellulolytic transcriptional activator Xyr1 (the ortholog of *A. niger* XlnR), suggested that this version of Cre1 may in fact act as a transcriptional activator (Rassinger et al., 2018). Similar truncations have not yet been studied in a wide variety of species and it is therefore not known whether this effect is conserved across fungal species.

CCR, mediated by CRE-1, has also been reported in *N. crassa* (Ebbole, 1998), and was for the first time studied in detail in a recent extensive study, in which the overall regulatory effects related to carbon utilization were assessed in this fungus (Wu et al., 2020). While the overall role of CRE-1 was similar to that described in our study, there were some notable differences. Promoter analysis together with DNA affinity purification sequencing (DAP-seq) analysis revealed a similar consensus binding site for CRE-1 (TSYGGGG), but only a small number of the *N. crassa* PCWD CAZy promoter regions (17%) contains a CRE-1 binding site compared to *A. niger* with 36% of PCWD CAZy promoters containing the adjacent SYGGGG motif. Similarly, binding sites were only detected for two transcriptional activators of *N. crassa*, while in contrast, a high percentage of the promoters of genes encoding putative sugar transporters contained CRE-1 binding sites and were confirmed by DAP-seq (42%). This suggests that a similar role for CreA across fungi does not exclude significant differences in how the effect is achieved. More detailed comparative studies will be needed using identical conditions to truly reveal the differences between the functional organization of CreA in fungal species, similar to what has been performed for XlnR previously (Klaubauf et al., 2014).

Although the deletion of *creA* increased the expression of key genes involved in plant biomass degradation and utilization at early stage of plant biomass conversion, no clear growth advantage was observed in the *A. niger creA* mutant compared to reference strain, which is in line with previous studies (Dowzer and Kelly, 1991, Ruijter et al., 1997). On the contrary, the *creA* mutant tends to grow slowly and abnormally with reduced sporulation on pure sugars and plant biomass substrates (Supplementary Fig. S7), as was also previously shown for *A. nidulans* (Khosravi et al., 2018). This could be due to a combination of an energetic burden of de-repressed non-essential systems, together with the downregulation of some important cellular processes by the deletion of CreA, such as protein translation, amino acid metabolism, and RNA binding (Fig. 1D). The abnormal fungal growth and changes of cellular processes in the *creA* mutant indicate that a temporary transcriptional repression mediated by CreA during the early stage of plant biomass conversion is crucial for maintaining the overall efficiency of fungal lignocellulose utilization. In addition, our findings support that CreA does not work alone, but acts in concert with specific transcriptional activators to precisely control the expression levels of their target genes. In the past decades, alleviating the CreA-mediated CCR has attracted broad interest in many bio-industries exploiting fungi as cell factories (Adnan et al., 2017). While many of those studies mainly emphasize removing repression through deleting the *creA* gene, our study suggests that simultaneous engineering of both carbon repression and transcriptional activation, while reducing the side effects of CreA deletion, should be taken into consideration in future strain engineering approaches.

In conclusion, our study provides strong indications for an important role of CreA in fungi under natural conditions, where low levels of free sugars are usually present. This likely explains the evolutionary conservation of CreA across the fungal kingdom, as loss of this regulator results in a reduction in efficiency of the overall physiology of fungi. This also implies that the different approaches for plant biomass conversion by fungi are mainly due to evolutionary changes in the transcriptional activators involved in the process, while the function of CreA has remained largely conserved. This is particularly the case for closely related species with highly similar genome content. However, it cannot be excluded that differences in the strength of the CreA-mediated repression in response to various monosaccharides exist across the fungal kingdom. Manipulation of CreA for industrial applications is not straight forward as demonstrated by the comparison of the *cre1* deletion and truncation in *T. reesei* (Rassinger et al., 2018), but has still enormous potential for generating strains with improved productivity. However, this requires a better understanding of the functioning of CreA and its molecular interaction with other regulators (Vinuselvi et al., 2012).

## CRediT authorship contribution statement

**Mao Peng:** Formal analysis, Data curation, Writing - original draft, Visualization. **Claire Khosravi:** Investigation, Formal analysis, Writing - original draft. **Ronnie J.M. Lubbers:** Investigation. **Roland S. Kun:** Investigation. **Maria Victoria Aguilar Pontes:** . **Evy Battaglia:** . **Cindy Chen:** . **Sacha Dalhuijsen:** Investigation. **Paul Daly:** . **Anna Lipzen:** Investigation, Formal analysis, Data curation. **Vivian Ng:** Investigation, Formal analysis, Data curation. **Juying Yan:** Investigation, Formal analysis, Data curation. **Mei Wang:** Investigation, Formal analysis, Data curation. **Jaap Visser:** . **Igor V. Grigoriev:** Investigation, Formal analysis, Data curation. **Miia R. Mäkelä:** Writing - review & editing. **Ronald P. de Vries:** Conceptualization, Supervision, Funding acquisition, Writing - review & editing, Project administration.

## Declaration of Competing Interest

The authors declare that they have no known competing financial interests or personal relationships that could have appeared to influence the work reported in this paper.
