# Supplementary material for: CreA-mediated repression of gene expression occurs at low monosaccharide levels during fungal plant biomass conversion in a time and substrate dependent manner
Source: Cell Surf. 2021 Mar 3;7:100050. doi: 10.1016/j.tcsw.2021.100050 (PMC7985698; doi:10.1016/j.tcsw.2021.100050)
Supplement: Supplementary data 1 [file mmc1.pdf]

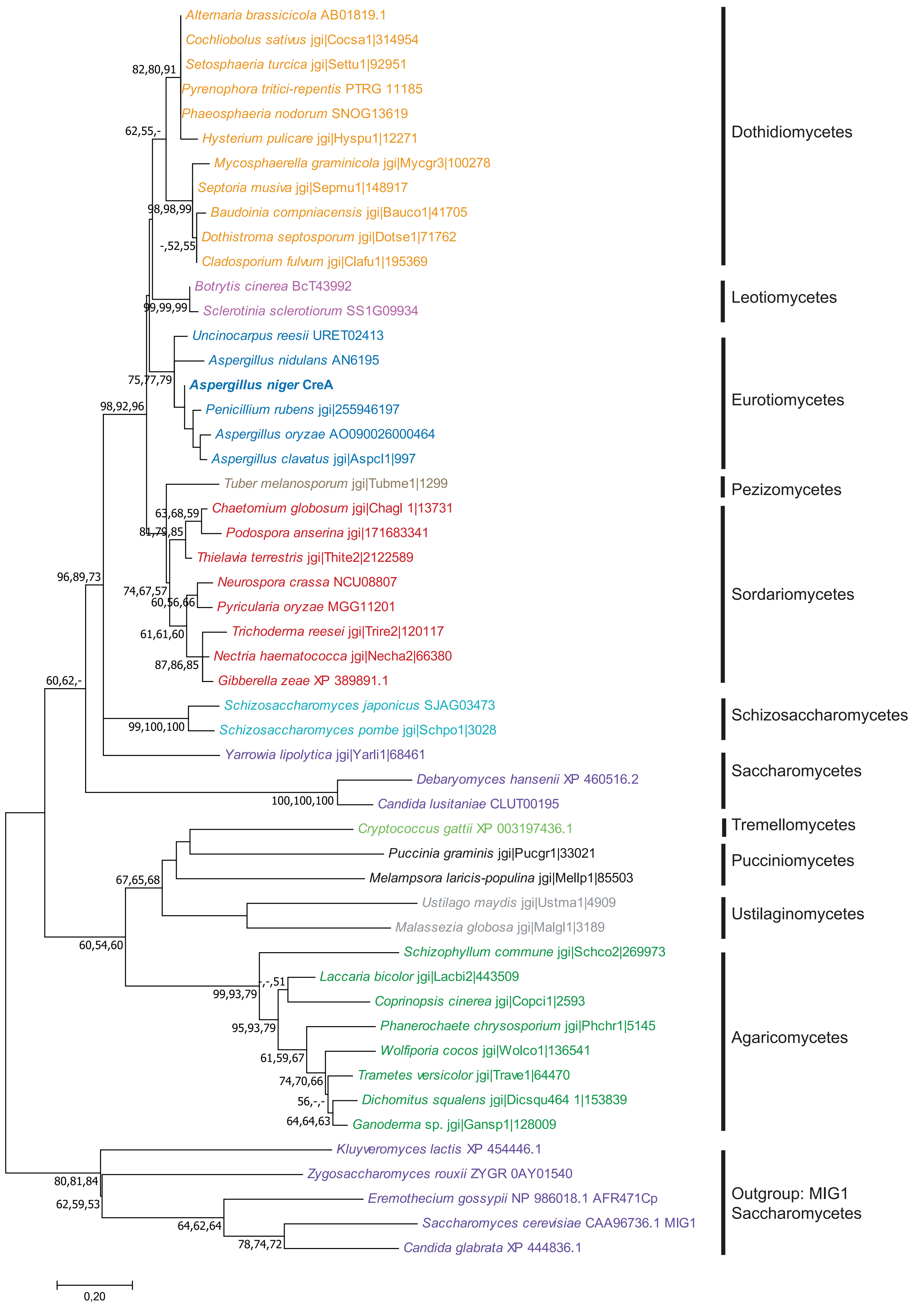

Supplemental Figure S1. Phylogenetic tree of the amino acid sequences of the CreA homologs of a representative set of fungal species across the fungal kingdom, demonstrating the conservation of CreA in fungi. The outgroup is MIG1 from *Saccharomyces cerevisiae*, which is a functional homolog of CreA, and several of its homologs. The tree is a representative Maximum Likelihood tree, generated using 500 bootstraps. Bootstrap values at the nodes represent the values of the Maximum Likelihood, Neighbor Joining and Minimal Evolution algorithms, respectively. Only bootstraps equal or higher than 50 are displayed. The colors reflect the fungal classes indicated on the right of the figure.

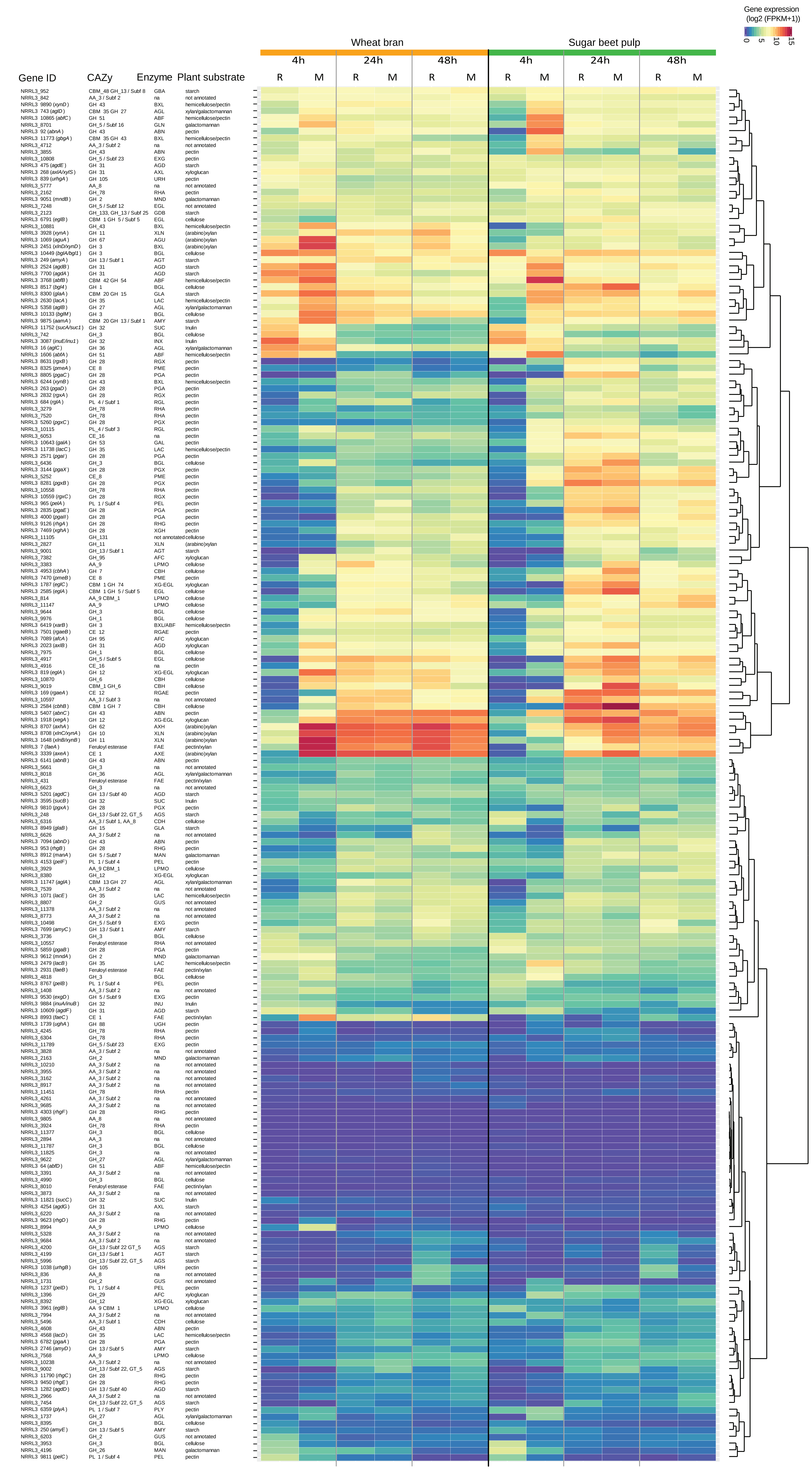

Supplemental Figure S2. Gene expression profile for 203 PCWD related CAZymes encoding genes in the *A. niger* Δ*creA* and reference strain. The average expression values (FPKMs) for each growth condition of reference and mutant were calculated and plotted on the heatmap. The sample names for reference and mutant are indicated with "R" and "M", respectively. PME = pectin methyl esterase, RGAE = rhamnogalacturonan acetyl esterase, ABN = endoarabinanase, ABF = α-arabinofuranosidase, LAC = β-galactosidase, EXG = exogalactanase, GAL = endogalactanase, PGA = endopolygalacturonase, GLN = exogalactanase, PGX = exopolygalacturonases, RHG = endorhamnogalacturonase, RGX = exorhamnogalacturonase, XGH = xylogalacturonase, PEL = pectin lyase, PLY = pectate lyase, RGL = rhamnogalacturonan lyase, RHA = α-rhamnosidase, UGH = unsaturated galacturonan hydrolase, AGS = α-glucan synthase, URH = unsaturated rhamnogalacturonan hydrolase, AMY = α-amylase, AGT = α-glucanotransferase, GBA = glycogen debranching enzyme, GLA = glucoamylase, AGD = α-glucosidase, INU = endoinulinase, LPMO = lytic polysaccharide mono-oxygenase, INX = exoinulinase, EGL = endoglucanase, XEG = xyloglucan-active endoglucanase, CDH = cellobiose dehydrogenase, AFC = α-fucosidase, AXL = α-xylosidase, XLN = endoxylanase, BGL = β-glucosidase, BXL = β-xylosidase, CBH = cellobiohydrolase, XBH = xylobiohydrolase, AXE = acetyl xylan esterase, AXH = arabinoxylan arabinofuranohydrolase, FAE = feruloyl esterase, AGU = α-glucuronidase, MAN = endomannanase, AGL = α-galactosidase, MND = β-mannosidase, GUS=β-glucuronidase, na= not annotated.

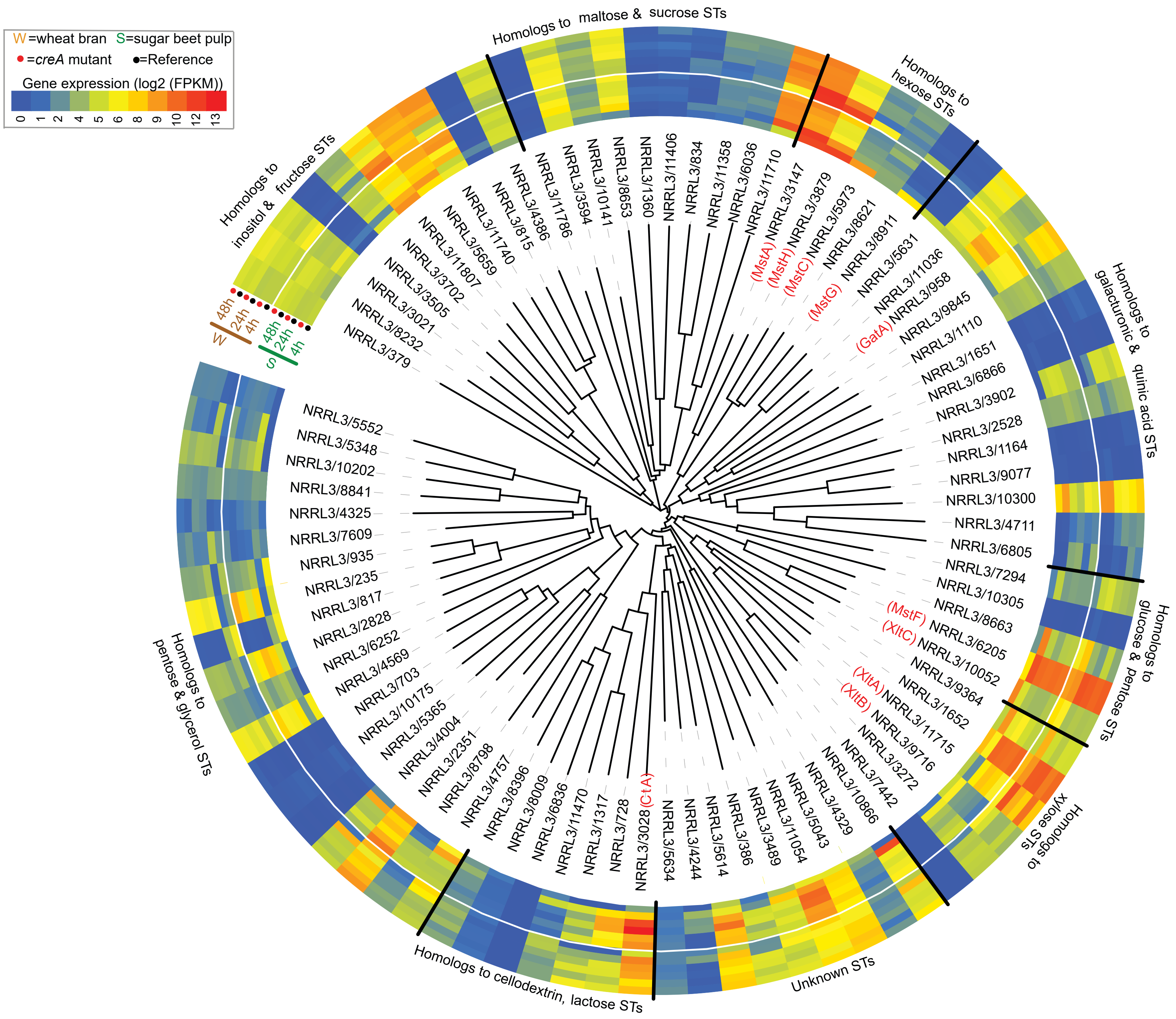

Supplemental Figure S3. Expression profile of sugar transporters between the *A. niger* Δ*creA* and reference strain. Sugar transporter (ST) genes and their phylogenetic analysis were derived from our previous publication (Peng et al., 2018a), and average expression values (FPKMs) of each ST at each growth condition were calculated and plotted on the heatmap. Ten previous experimentally characterized STs are highlighted with red fonts.

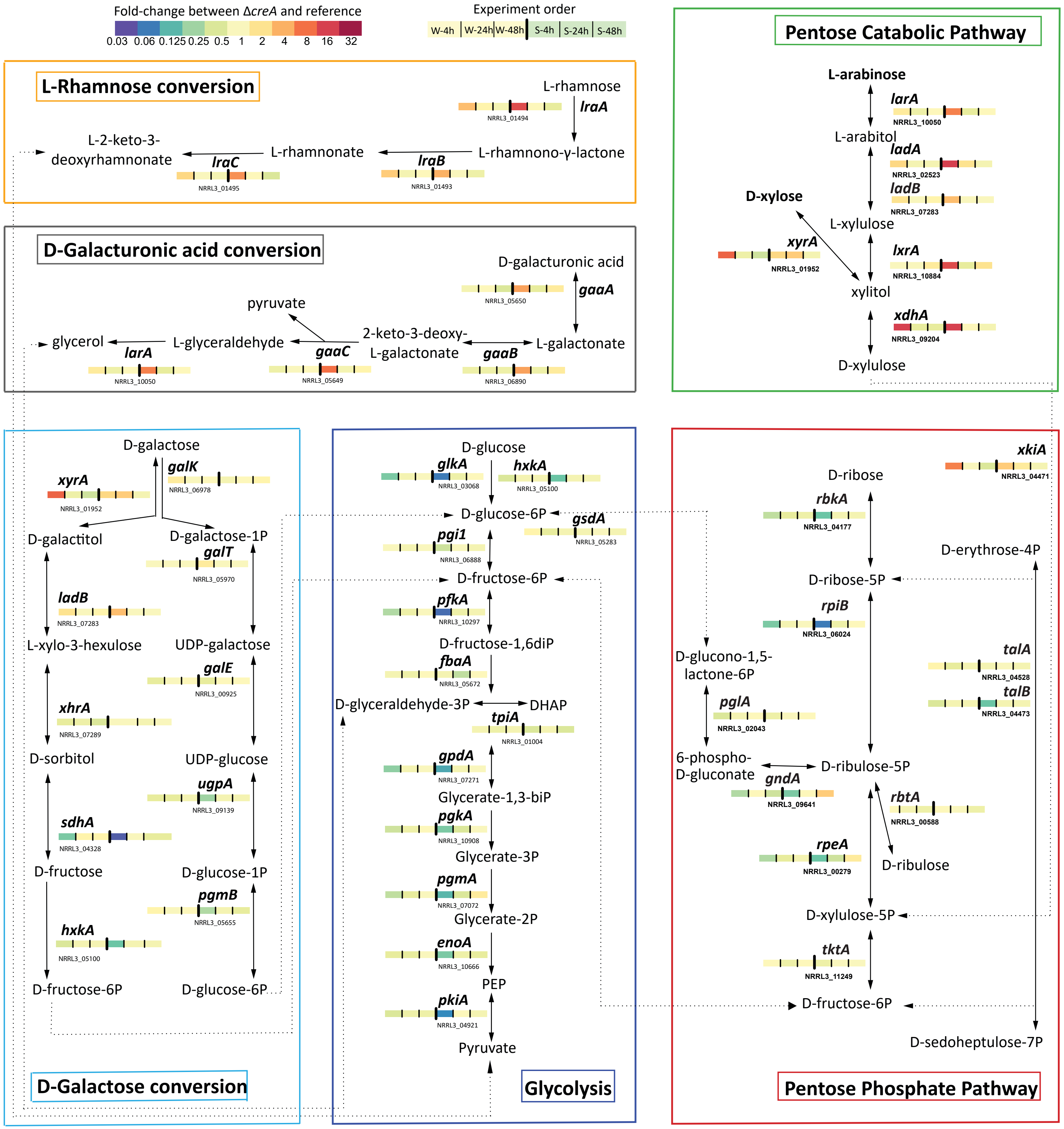

Supplemental Figure S4. Selective regulation of different sugar catabolic pathways by CreA in *A. niger*. Carbon catabolic enzymes and pathways were retrieved from a previous study (Aguilar-Pontes et al., 2018). The fold changes of gene expression between Δ*creA* and reference strain from lower to higher expression are indicated by colors from blue to red. The ‘W’ and “S” indicate fungal growth substrates with wheat bran and sugar beet pulp, respectively.

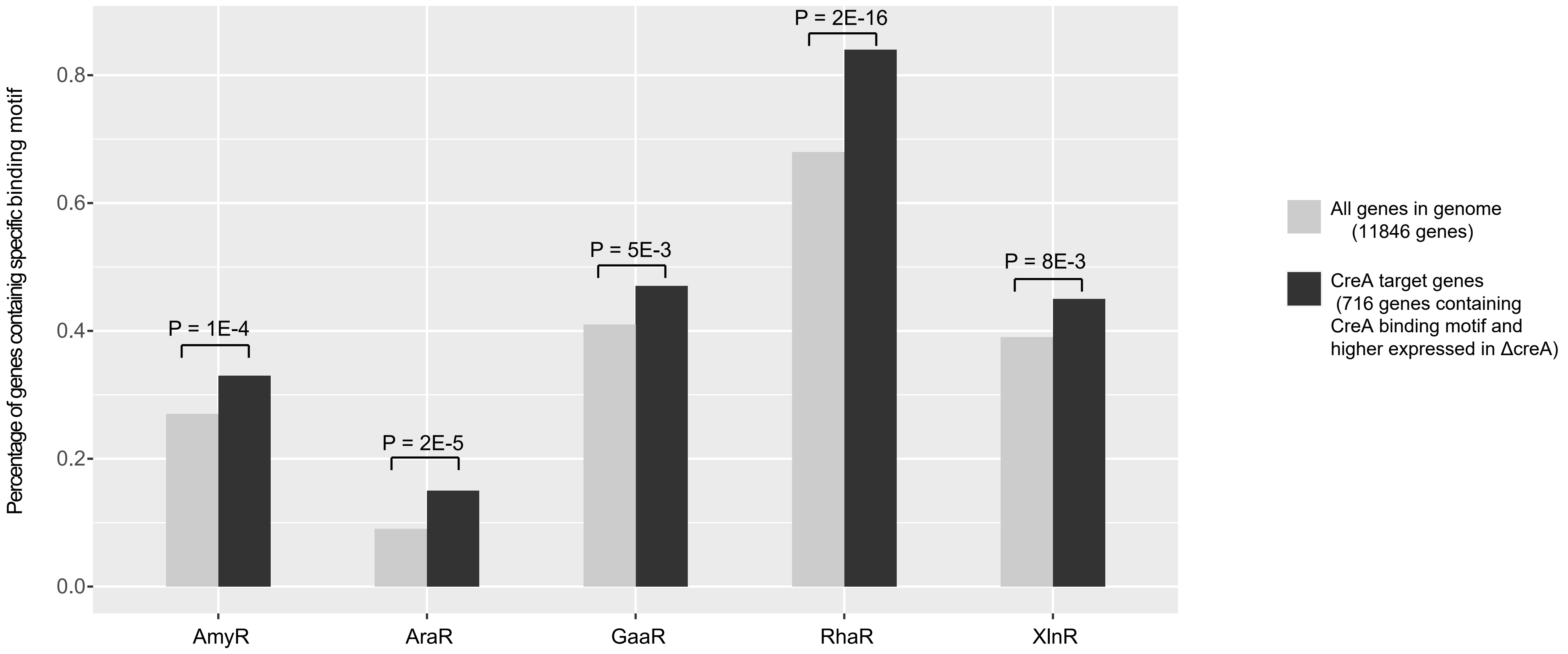

Supplemental Figure S5. Overrepresentation of other transcription factor's binding motif in CreA target genes. The bar chart showed the comparison of percentage of genes containing binding motifs of XlnR, AraR, RhaR, GaaR or AmyR between CreA target genes and all genes in genome. P-values was calculated with the Fisher's exact test.

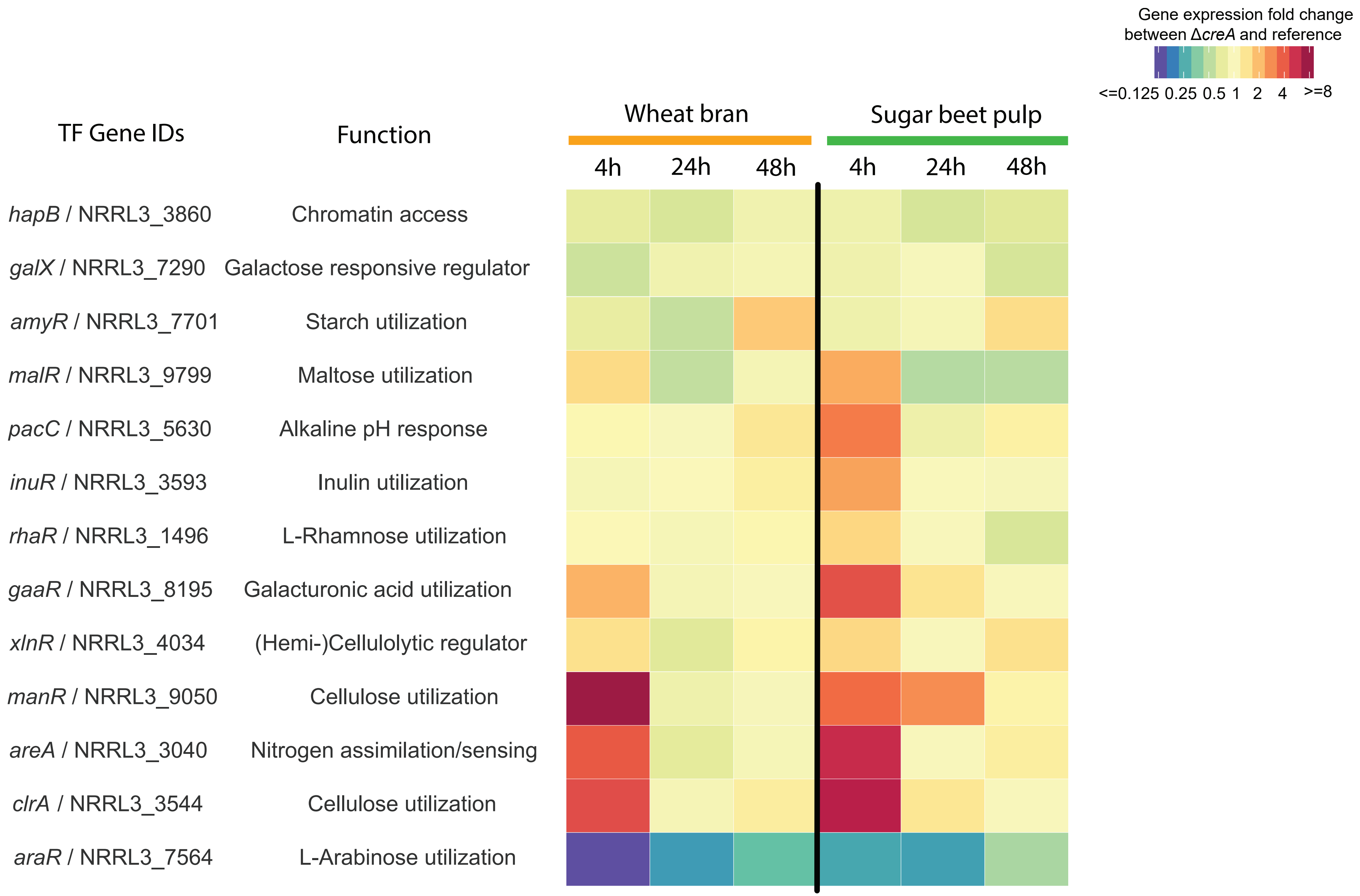

Supplemental Figure S6. Transcriptomic changes of plant biomass conversion related transcription factor encoding genes between *A. niger* Δ*creA* and reference strain. The colors from blue to red indicate genes from lower to higher expression in Δ*creA* compared to reference strain. Only the genes with fold change≥1.5 and adjusted P-value < 0.01 in the comparison between the reference and Δ*creA* are shown in the figure.

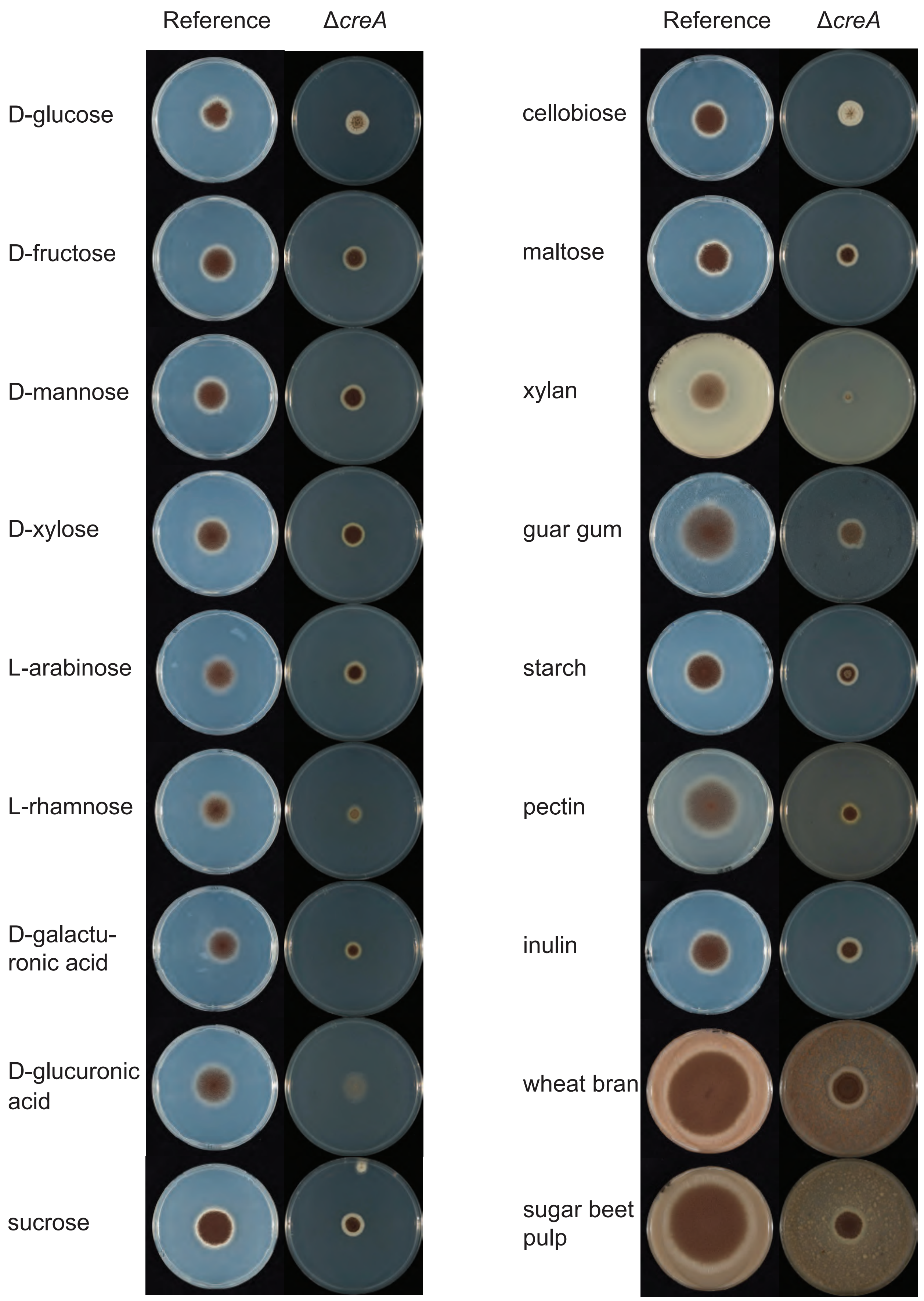

Supplemental Figure S7. Comparison of the growth of *A. niger* reference and Δ*creA* strain on a range of carbon sources. The growth of the Δ*creA* is reduced on all carbon sources, but the extent of this depends on the carbon source. In addition, sporulation is reduced on a number of carbon sources for the Δ*creA*.
